# Technology: A Friend or Foe in the Context of Person-Centeredness?

**DOI:** 10.1093/geroni/igaf122.1382

**Published:** 2025-12-31

**Authors:** Sheryl Zimmerman

**Affiliations:** University of North Carolina at Chapel Hill, Chapel Hill, North Carolina, United States

## Abstract

As is true for all cohorts, technology holds promise to improve quality of care and quality of life for older adults, their families, and their care providers. At the same time, technology can negatively affect quality of care and life, such as by increasing burden on record-keeping with no demonstrable benefit to care and outcomes. More so, while literature is replete with studies about innovative technologies, it rarely examines them using a lens of person-centeredness, which emphasizes how technology serves an individual’s interests, values, goals of care, and needs. This presentation will provide examples from clinical trials in long-term care illustrating how technology can be either agnostic or responsive to person-centeredness, and key issues identified through related discussion groups with experts in long-term care services and supports, technology design and evaluation for older persons, and aging-related health and function. Challenges identified from the discussion groups include that (1) technology can both facilitate and impede person-centeredness; (2) designing technologies in a person-centered manner requires participation of potential users in every stage of development; (3) ease of use and adoption of a new technology should be at the forefront in advanced design and implementation planning; and (4) implementation of new technologies should progress in a manner that reduces racial, geographic, and income-related disparities. Potential mitigating factors include involving users in design; considering return on investment and value-based measures; understanding the interaction between care providers and technology; and making technology more equitable. These topics will be discussed and audience perspectives invited.

